# Comorbidities in SARS-CoV-2 Patients: a Systematic Review and Meta-Analysis

**DOI:** 10.1128/mBio.03647-20

**Published:** 2021-02-09

**Authors:** Wern Hann Ng, Thomas Tipih, Nigel A. Makoah, Jan-G Vermeulen, Dominique Goedhals, Joseph B. Sempa, Felicity J. Burt, Adam Taylor, Suresh Mahalingam

**Affiliations:** a Emerging Viruses, Inflammation and Therapeutics Group, Menzies Health Institute Queensland, Griffith University, Gold Coast, Southport, QLD, Australia; b Division of Virology, Faculty of Health Sciences, University of the Free State, Bloemfontein, South Africa; c Division of Virology, National Health Laboratory Service, Bloemfontein, South Africa; d Department of Biostatistics, Faculty of Health Sciences, University of the Free State, Bloemfontein, South Africa; e DST-NRF Centre of Excellence in Epidemiological Modelling and Analysis (SACEMA), Stellenbosch University, Stellenbosch, South Africa; Johns Hopkins Bloomberg School of Public Health

**Keywords:** COVID-19, comorbidity, SARS-CoV-2

## Abstract

Severe acute respiratory syndrome coronavirus 2 (SARS-CoV-2) has rapidly spread across the globe at unprecedented speed and is showing no signs of slowing down. The outbreak of coronavirus disease 2019 (COVID-19) has led to significant health burden in infected patients especially in those with underlying comorbidities. The aim of this study was to evaluate the correlation between comorbidities and their role in the exacerbation of disease in COVID-19 patients leading to fatal outcomes. A systematic review was conducted using data from MEDLINE, Scopus, Web of Science, and EMBASE databases published from 1 December 2019 to 15 September 2020. Fifty-three articles were included in the systematic review. Of those 53 articles, 8 articles were eligible for meta-analysis. Hypertension, obesity, and diabetes mellitus were identified to be the most prevalent comorbidities in COVID-19 patients. Our meta-analysis showed that cancer, chronic kidney diseases, diabetes mellitus, and hypertension were independently associated with mortality in COVID-19 patients. Chronic kidney disease was statistically the most prominent comorbidity leading to death. However, despite having high prevalence, obesity was not associated with mortality in COVID-19 patients.

## Abstract (precis)

COVID-19 has plagued the world since it was first identified in December 2019. Previous systematic reviews and meta-analysis were limited by various factors such as the usage of non-peer reviewed data and were also limited by the lack of clinical data on a global scale.

## INTRODUCTION

Coronaviruses (CoVs) are RNA viruses with a large nonsegmented genome and can infect both animals and humans (1). CoV infections can cause respiratory distress, gastrointestinal and hepatic diseases, and neurological complications (2). With the recent emergence of severe acute respiratory syndrome coronavirus 2 (SARS-CoV-2), responsible for the current coronavirus disease 2019 (COVID-19) pandemic, there are now seven coronaviruses known to infect humans (3–5). First identified in Wuhan, China, SARS-CoV-2 has since spread rapidly around the globe with over 210 countries and territories reporting infections. The global new COVID-19 cases and deaths are soaring. Increasing numbers of cases and deaths are being reported weekly since early October 2020. The numbers peaked in the second week of November 2020 with almost 4 million new cases and 60,000 new deaths recorded. As of 13 December 2020, SARS-CoV-2 is known to have infected over 70.4 million individuals with more than 1.5 million associated deaths reported (6). Fortunately, through a global effort, COVID-19 vaccines are now entering the market to slow the spread of COVID-19, with the vaccines developed by Pfizer-BioNTech and Moderna both reporting greater than 94% efficacy in clinical trials (7). The Pfizer-BioNTech COVID-19 vaccine has been approved for use in the United States, Canada, and United Kingdom, and priority is being given to people over 80 years of age and health care workers (8). On 18 December 2020, the U.S. Food and Drug Administration issued an emergency use authorization for Moderna’s vaccine (7, 9, 10). However, caution is still warranted, as it is still unknown whether these vaccines will provide long-term protection.

There are multiple risk factors that are associated with COVID‐19. For example, the male population has a higher rate of SARS-CoV-2 infection compared to females (11, 12). Studies have shown that a higher incidence of severe and fatal COVID-19 is observed with increasing age (13), and it is speculated that this phenomenon is partly attributed to preexisting comorbid conditions (14). To date, there are several systematic reviews being published regarding the effect of comorbidities on prognosis of COVID-19 patients. However, much of the previous data analysis is limited by factors such as incomplete prevalence reporting due to the use of non-peer reviewed data and only using data from China (15, 16). This limits the conclusions that can be drawn from these early studies, particularly given the global reach of the SARS-CoV-2 pandemic.

As the pandemic has progressed, an increasing amount of clinical data has been made available from around the world. The aim of this study is to present an updated systematic review on the influence of comorbidities on the exacerbation of COVID-19. Here we analyze the most recent data available in the literature to gain better insights into the development of COVID-19 and severe forms of the disease resulting in death to aid the development of strategies to better manage SARS-CoV-2-infected patients.

## RESULTS

### Study selection.

A total of 4,266 articles were identified using the search strategy employed in MEDLINE, Scopus, Web of Science, and EMBASE databases. A total number of 3,143 duplicates and irrelevant articles were removed, bringing the number of articles screened for title and abstract to 1,123. Subsequently, 778 articles were excluded after title and abstract screening, resulting in 345 studies which were subjected to full-text screening. Fifty-three studies matched our predetermined inclusion and exclusion criteria. These studies included a total number of 375,859 participants from 14 countries, namely, Brazil, China, India, Iran, Italy, Mexico, Oman, Saudi Arabia, South Korea, Spain, Turkey, Uganda, United Kingdom, and United States (see Table S1 in the supplemental material).

10.1128/mBio.03647-20.1 TABLE S1 Characteristics of included studies. Data included in this table, when available, included the title, country, age, number of participants, and the type of analysis applied. Download Table S1, DOCX file, 0.05 MB. Copyright © 2021 Ng et al. 2021 Ng et al. This content is distributed under the terms of the Creative Commons Attribution 4.0 International license.

Among the selected articles, two articles specifically studied adult patients (17, 18), while three articles focused on pediatric patients (19–21). The remaining 49 articles did not impose any age limitations in their respective studies. However, despite SARS-CoV-2 testing in all patients, these studies did not identify positive samples in patients with a mean age of <18 years, which aligns with previous observations suggesting age plays a role in the severity of SARS-CoV-2 infection (13, 14, 22, 23). The three most prevalent comorbidities were hypertension, obesity, and diabetes amounting to 80,093 (21.3%), 68,935 (18.3%), and 67,954 (18.1%) patients, respectively. The comorbidities were reported as defined, respectively, from extracted studies.

Clinical outcomes reported in study participants included mortality, severe disease, hospitalization, length of hospitalization, the need for intubation, and development of acute respiratory distress syndrome. In one study, the final clinical outcome of patients was not reported (24).

The majority of studies included in the analysis did not report the status of the comorbidities in patients. Two articles investigated the role of cancer in COVID-19 patients. One study included 12 participants of which 7 had received anticancer treatments a month prior to the study (25), while in another Italian cohort, active cancer independently predicted mortality in COVID-19 patients (26). Chronic kidney disease stage was reported in two studies (27, 28), and later disease stages were not associated with a worse prognosis in study participants from Iran (27), while the effect of higher chronic kidney disease states on prognosis was not evaluated in a Spanish study (28). In a cohort of COVID-19 patients with chronic kidney and end-stage renal diseases, only the former was reported as an independent predictor of the requirement of intensive care unit (ICU) level care (29).

One study categorized obese participants into two groups, having a body mass index (BMI) of 30 to 40 kg/m^2^ or BMI of ≥40 kg/m^2^. Having a BMI above 40 kg/m^2^ and being younger than 50 years was independently associated with mortality (30). A study by Docherty et al. (31) grouped diabetic patients into those with and without complications. However, the individual effect of each of the groups on patient outcome was not reported. None of the studies included in the analysis evaluated the role of antidiabetic therapy on SARS-CoV-2 outcome in patients.

### Mortality.

**FIG 1:**
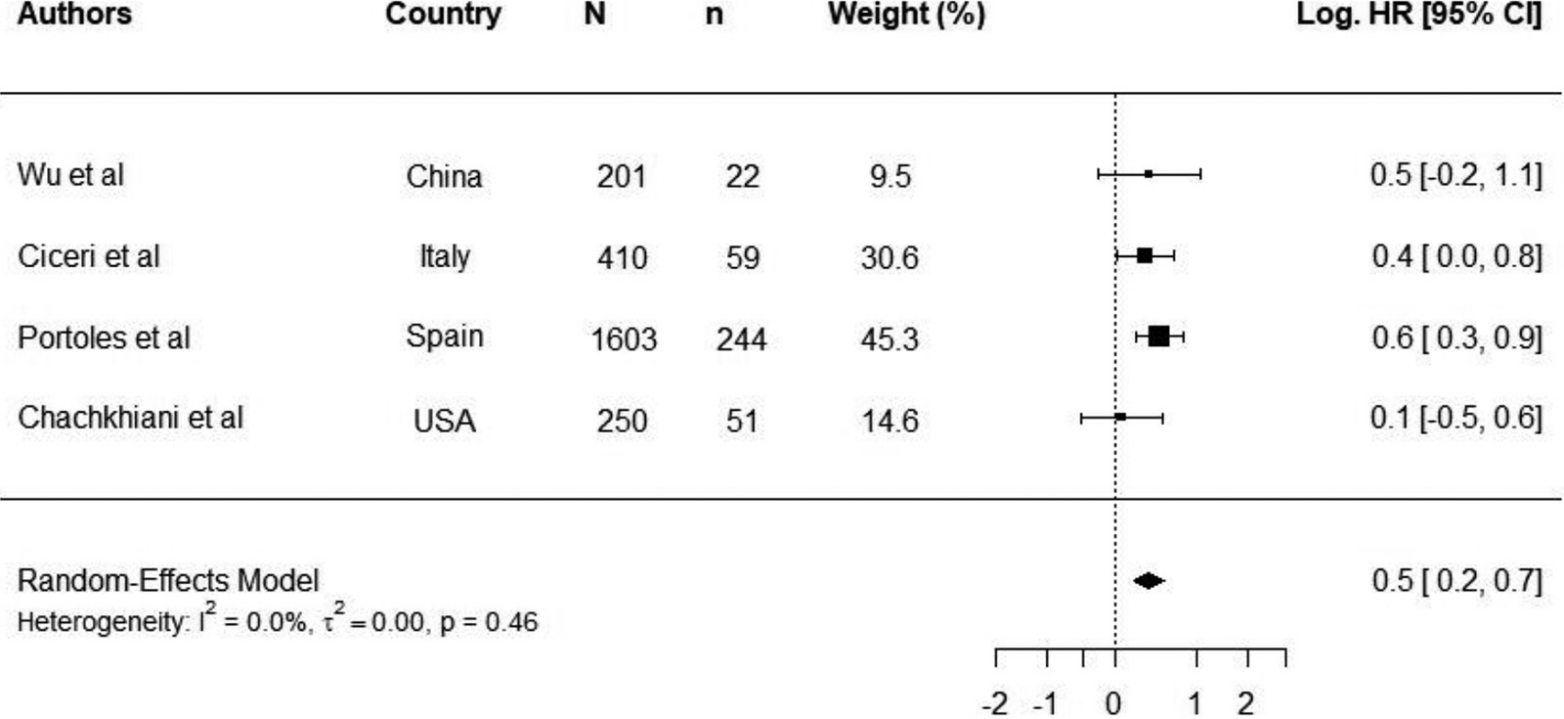
Log hazards ratios of COVID-19-related mortality in patients with diabetes. Log.HR was calculated from four independent studies (Wu et al. [53], Ciceri et al. [26], Portoles et al. [28], and Chachkhiani et al. [32]). N is the study size, n is the number of participants with comorbidity, and Log.HR is the natural logarithm of hazard ratio.

**FIG 2:**
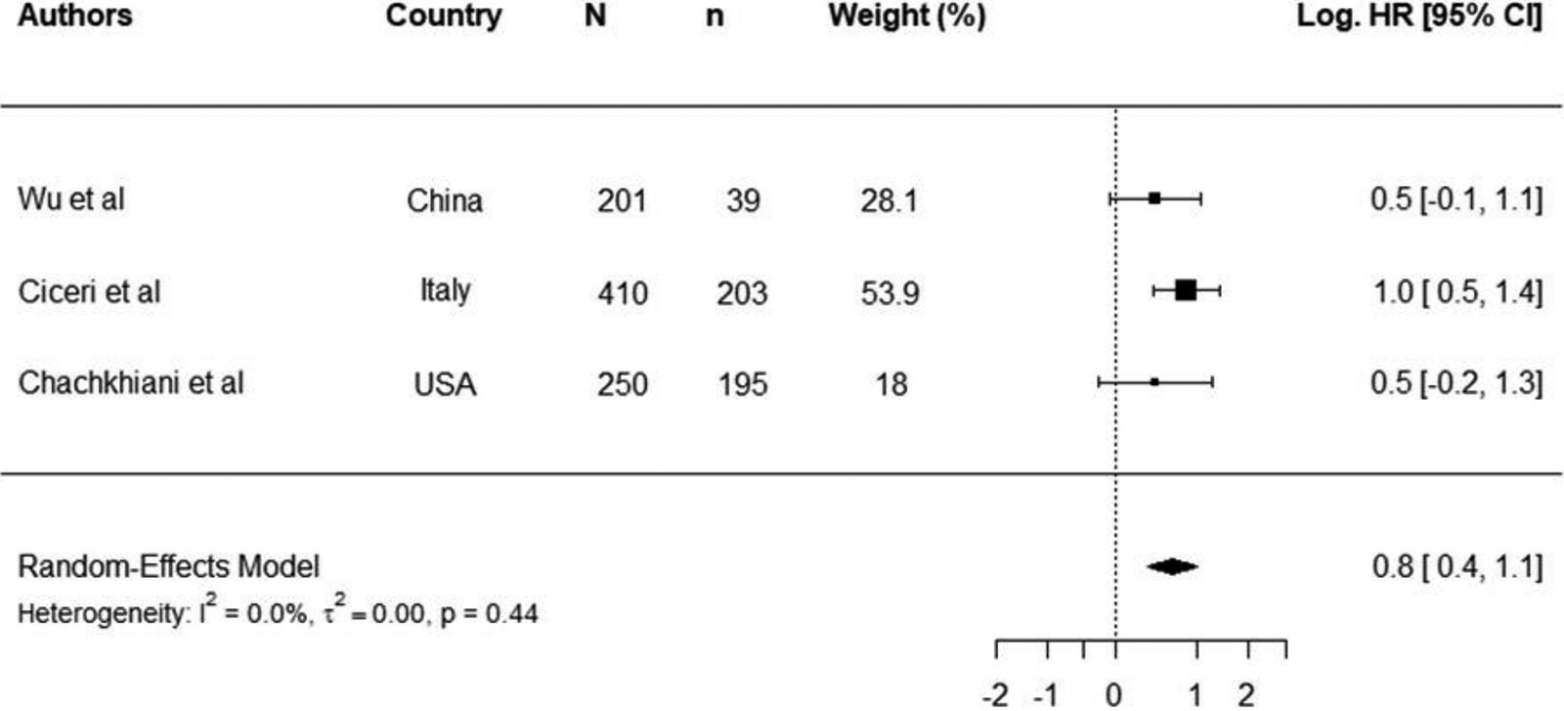
Log hazards ratios of COVID-19-related mortality in patients with hypertension. Log.HR was calculated from three independent studies (Wu et al. [53], Ciceri et al. [26], and Chachkhiani et al. [32]). N is the study size, n is the number of participants with comorbidity, and Log.HR is the natural logarithm of hazard ratio.

Thirty studies reported mortality as a clinical outcome in COVID-19 patients (17, 18, 26–28, 30–54). Independent risk factors for mortality were reported in 22 studies, and these risk factors included hypertension, obesity, diabetes, renal disease, chronic obstructive pulmonary disease (COPD), neurological disease, and cardiovascular disease (18, 26, 30–37, 40, 42–52). Eight studies were selected for meta-analysis of cancer (36, 39), chronic kidney diseases (26, 28, 39), diabetes (26, 28, 32, 53), hypertension (26, 32, 53), and obesity (28, 37) as comorbidities. Meta-analysis results for comorbidities in which three or more studies were included in the analysis are shown in Fig. 1 and 2. By virtue of having larger sample sizes, we believe meta-analysis results for diabetes and hypertension are more reliable.

The overall natural logarithm of odds ratio (log.OR) of mortality in cancer patients was 0.49 (95% confidence interval [95% CI], 0.01 to 0.97), which translates to an OR of 1.63 (95% CI, 1.01 to 2.00). The results indicate a significant 63% increased odds of COVID-19-related mortality in patients with cancer.

The overall natural logarithm of hazard ratio (log.HR) of mortality in patients with chronic kidney diseases was 1.28 (95% CI, 0.89 to 1.67), which translates to an HR of 3.61 (95% CI, 2.45 to 5.32). The results indicate a significant 3.6 times increased hazard of COVID-19-related mortality in patients with chronic kidney diseases.

The overall log.HR of mortality in patients with diabetes was 0.46 (95% CI, 0.24 to 0.67), which translates to an HR of 1.94 (95% CI, 1.54 to 2.46) (Fig. 1). The results indicate a significant 94% increased hazard of COVID-19-related mortality in patients with diabetes.

The overall log.HR of mortality among patients with hypertension and nonhypertensive patients was log.HR 0.8 (95% CI, 0.40 to 1.10) (Fig. 2), which translates to a HR of 2.10 (95% CI, 1.50 to 2.90). The results imply that the hazard of COVID-19 mortality was increased 2.1 times in patients with hypertension, indicating a significant effect on mortality in COVID-19 patients.

The overall log.HR of COVID-19 mortality among obese patients was 0.45 (95% CI, −0.03 to 0.94) which translates to an HR of 1.58 (95% CI, 0.96 to 2.57). The results indicate a nonsignificant 58% increased hazard of COVID-19 mortality in patients with obesity. Overall, there was low heterogeneity in all meta-analyses, which implied that performing a random-effects meta-analysis was unnecessary.

### Severe disease.

Ten studies reported severe disease as an outcome in COVID-19 patients (19, 54–62). The definition of severe disease, however, varied among studies. Independent predictors of severe infection were described in three studies, and these predictors included neurological disease, neoplastic disease, arterial hypertension (59), obesity (55), and diabetes mellitus (57). One study reporting severe disease as an outcome specifically targeted pediatric patients with a median age of 16 years and a total of 407 patients. Critical illness was linked with increasing age, longer duration of symptoms, and lower oxygen saturation on presentation. Twenty-four of the children tested positive for SARS-CoV-2, and 19 of them required hospitalization. Of those 19 patients, 7 were critically ill, with 4 requiring intubations. Comorbidities were present in two out of the four intubated patients, and one of them died from a sudden cardiac arrest (19).

### Severe outcome.

A composite severe outcome in COVID-19 patients was reported in three studies (25, 63, 64). Of the three studies, only cancer with an OR of 6.51 (95% CI, 1.72 to 24.64; *P* = 0.006) was reported as an independent risk factor for a severe outcome in a study with participants from China (25). In a Spanish cohort of 456 SARS-CoV-2-positive rheumatic and nonrheumatic patients, connective tissue disorder with an OR of 1.64 (95% CI, 1.02 to 2.66; *P* = 0.042), obesity with an OR of 1.78 (95% CI, 1.13 to 2.81; *P* = 0.013), diabetes with an OR of 1.81 (95% CI, 1.11 to 2.95; *P* = 0.018), hypertension with an OR of 2.60 (95% CI, 1.72 to 3.94; *P* < 0.001), heart failure with an OR of 3.49 (95% CI, 2.21 to 5.51; *P* < 0.001), and lung disease with an OR of 2.15 (95% CI, 1.34 to 3.45; *P* = 0.001) could be reported as risk factors for a severe outcome only in bivariate analysis.

### Hospitalization.

Hospitalization as an outcome was reported in nine studies (21, 48, 51, 65–70). The criteria for hospitalization are expected to vary in different countries and studies. Factors independently predicting hospitalization were identified in five studies (48, 51, 66–68), and commonly identified comorbidities included diabetes, chronic kidney diseases, obesity, smoking, and COPD. In a study by van Gerwen et al. (51), diabetes was associated with an increased risk of mechanical ventilation with an OR of 1.35 (95% CI, 1.08 to 1.69) in hospitalized patients, and an observational longitudinal study of COVID-19 patients with autoimmune inflammatory rheumatic diseases identified the presence of a systemic autoimmune condition (OR, 3.55; 95% CI, 1.30 to 9.67; *P* = 0.01) as a risk factor for hospitalization (66). Although the immunosuppressant medication for autoimmune patients may predispose patients to severe disease, rheumatic patients enrolled in the study were mostly elderly and presented with comorbidities (66). Despite a 14% prevalence of asthma in one study, the comorbidity was not associated with an increased risk of hospitalization with a relative risk of 0.96 (95% CI, 0.77 to 1.19; *P* = 0.71) (65). In a cohort of COVID-19 patients from metropolitan Detroit (MI, USA), severe obesity with an OR of 2.0 (95% CI, 1.4 to 3.6; *P* = 0.02) and chronic kidney disease with an OR of 2.0 (95% CI, 1.3 to 3.3; *P* = 0.006) were independently associated with intensive care stay in hospitalized patients (29). Additionally, severe obesity with an OR of 3.2 (95% CI, 1.7 to 6.0; *P* < 0.001), chronic kidney disease with an OR of 2.4 (95% CI, 1.4 to 4.2; *P* = 0.001), and cancer with an OR of 2.5 (95% CI, 1.2 to 5.0; *P* = 0.01) were independently associated with the need for mechanical ventilation (29).

In a cohort of COVID-19 patients with neurological complaints, altered mental status was identified as an independent predictor of prolonged hospital stay with an OR of 1.6 (95% CI, 1.1 to 2.5; *P* = 0.01) and the requirement for intubation with an OR of 4.9 (95% CI, 2.6 to 9.4; *P* < 0.0001) (32).

## DISCUSSION

The association between comorbidities and their role in the exacerbation of COVID-19 in patients leading to death is evaluated in this study, using published results from large cohort data from across the globe. Our study identified hypertension as the most common comorbidity in COVID-19 patients followed by obesity and diabetes. This partially resonates with earlier publications on the clinical characteristic and frequency of comorbidities in SARS-CoV-2-infected patients where circulatory disease (including hypertension and coronary artery diseases) was reported to be the highest (71–73). In the meta-analysis, we demonstrated that chronic kidney disease, hypertension, and diabetes mellitus were associated with COVID-19 mortality. The latter two are among the most prevalent comorbidities in COVID-19 patients and were associated with a higher risk of fatality and often coexist as multiple comorbidities along with obesity (51). In patients with chronic kidney disease, the risk of in-hospital mortality in COVID-19 patients appears higher in cases with end-stage renal disease compared to chronic renal disease (50). However, in one study, only chronic kidney disease was associated with the requirement of ICU level care in COVID-19 patients (29). Recently, a study reported a staggering 49% of cumulative incidence of thrombotic complications in COVID-19 patients (74). COVID-19 is associated with increased clot strength, platelet fibrinogen contribution to clot strength, elevated d-dimer levels, and hyperfibrinogenemia (75). Hence, the association of severe outcomes in patients with hypertension and diabetes may be partially explained by the increased incidence of thrombotic complications as it is already established that patients with hypertension and diabetes have elevated risk of thrombotic events (76, 77).

Further investigation to explain the higher prevalence of reported comorbidities in COVID-19 patients may focus on the SARS-CoV-2 cell entry mechanism. Similarly to SARS-CoV-1, SARS-CoV-2 contains a receptor-binding domain (RBD) that recognizes angiotensin-converting enzyme 2 (ACE2) as its receptor with a higher binding affinity compared to SARS-CoV-1 (78). ACE2 receptor is commonly identified in the epithelial cells of the lungs, intestine, kidney, and blood vessel (79). Diabetic patients are frequently prescribed with thiazolidinedione, a type of oral hypoglycemic used to lower blood glucose level. Thiazolidinedione is reported to increase the expression of ACE2 (80). ACE inhibitors (ACE-I) and angiotensin II type 1 receptor blockers (ARBs) are also frequently used as a treatment strategy to treat diabetes and hypertension (81). Consequently, increased expression of ACE2 may promote the internalization of SARS-CoV-2, which in turn may increase the chances of developing COVID-19 or a severe form of the disease. However, despite clear evidence that ACE2 is the main receptor for COVID-19 (82), several studies have encouraged the continued use of ACE-I and ARB medications in patients with diabetes or circulatory diseases as use of these medications is not associated with severe COVID-19 and may even be beneficial in reducing disease severity in these patients (57, 60, 83, 84).

Chronic medical conditions, similar to infectious diseases, often present with inflammation and weakened innate immune responses in affected individuals. This may predispose those individuals to infections and disease complications (15). The high prevalence of fatal cases among COVID-19 patients with hypertension and diabetes as comorbidity could be due to the induction of cytokine storm. Cytokine storms resulting in hyperinflammation are the hallmarks of severe SARS-CoV-2 infection (85). Besides participating in host defenses against infectious agents, inflammation contributes to the pathogenesis of many chronic disorders such as diabetes mellitus, cardiovascular disease, and cancer (86). Inflammation is therefore regarded as a link between pathogens and chronic disorders. Metabolic inflammation as a consequence of hypertension and diabetes is also known to compromise the immune system, thereby weakening the host’s ability to fend off infections. Hypertension and diabetic patients are commonly reported to have weakened immunological function arising from reduced macrophage and lymphocyte activity which could predispose individuals to infections, especially those infections for which cell-mediated immunity constitute an important host defense (87). Similarly, the use of immunosuppressive drugs for the treatment of autoimmune diseases and cancer in patients also suffer from weakened immunological functions. This may explain the increased risk of hospitalization observed in these patients particularly in those that are critically ill (66, 88). Thus, patients with an underlying comorbidity, particularly cancer, hypertension, and diabetes, face a higher risk of mortality as evidenced by our findings.

Obesity was not identified as an independent predictor of mortality in our meta-analysis. However, these results should be interpreted with caution, since only two studies were included in the analysis. Obesity is unanimously accepted to be a common morbidity among critically ill patients, including its association with increased mortality (89–92). Unlike diabetes and hypertension, obesity does not directly influence the expression of ACE2. Rather, obese patients have a larger amount of ACE2-expressing adipose tissue in the body which indirectly results in an increased amount of ACE2-expressing cells (93). Other factors put forward to explain COVID-19 severity in obese individuals include abnormal cytokine and complement production leading to the reduction in the activity processes that inhibits acute inflammation (94, 95). Increased risk of blood clotting due to obesity has also been proposed to play a role (95, 96). Prolonged viral shedding is also associated with obesity in SARS-CoV-2-infected patients, which may also contribute to COVID-19 aggravation leading to mortality (97). Moreover, managing obese patients requiring intensive care presents with practical difficulties (which affect prognosis) such as the need for bariatric beds, challenging intubations, weight limitations on imaging machines impacting imaging diagnosis and the fact that obese patients are more inclined to take prone positions promoting ventilatory dysfunction (98).

There are several limitations to our findings. Our search obtained non-English articles which were excluded. In addition, many studies failed to report bivariate analysis for the investigated comorbidities leading to only eight included studies in the meta-analysis. It should also be noted that patients with chronic kidney diseases, hypertension, diabetes, and cancer are already at increased risk of mortality in the absence of COVID-19 (99–102). These limitations may contribute to the overestimation or underestimation of comorbidity and its role in the exacerbation of SARS-CoV-2 leading to fatal outcomes. We attempted to but may not have fully controlled for heterogeneity which may introduce bias into our findings, as patients included in our study are of various ages, ethnicities, and living conditions among other factors. Furthermore, some studies failed to report the type of intervention applied, such as the type of medication prescribed to affected patients, which could lead to a various number of reported casualties. In this study, data also suggested that several patients had more than one comorbidity but did not confirm whether the risk increased with the number of comorbidities. SARS-CoV-2 is claiming more lives daily, and the number of cases is still increasing. From our data, patients with comorbidities should be given prompt care to avoid complications.

Our study identifies hypertension to have the highest prevalence as a comorbidity in COVID-19 patients. Patients with chronic kidney diseases appear to face a higher risk of fatality while hypertension, diabetes, and cancer are found to significantly exacerbate the severity of COVID-19 in patients resulting in death. Evidence presented in this study will help to determine high-risk COVID-19 patients so that appropriate measures can be taken to mitigate the number of fatalities in the COVID-19 pandemic.

## MATERIALS AND METHODS

Preferred Reporting Items for Systematic Review and Meta-analysis (PRISMA) guidelines were used in this review (103). This study aims to report the influence of comorbidities and its role in exacerbation of COVID-19 leading to fatality in infected patients.

### Ethical approval.

Ethical clearance for the study was obtained from the Health Sciences Research Ethics Committee (HSREC) (ethics number UFS-HSD2020/1150/2807/-0001) at the University of the Free State.

### Search strategy.

The MEDLINE, Scopus, Web of Science, and EMBASE databases were searched for articles published from 1 December 2019 to 15 September 2020. The search strategy employed was as follows: (SARS-CoV-2 or COVID-19 or 2019-nCoV) AND (underlying or comorbid* or comorbid*) AND (prognos* or outcome*). The search was restricted to include only articles printed in English.

### Study selection.

**FIG 3:**
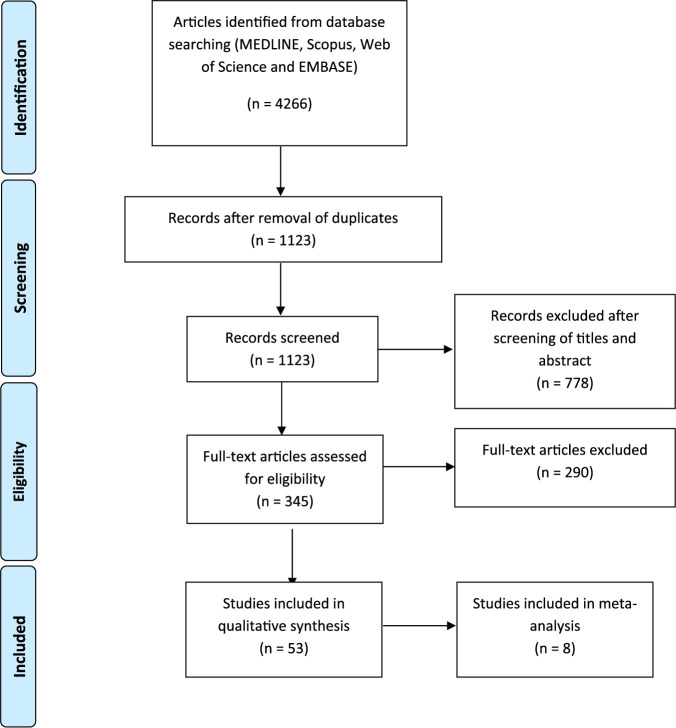
Flow diagram outlining study selection, eligibility, and inclusion in meta-analysis.

Study selection was conducted by authors, W.H.N. and T.T. independently. Identified articles were pooled together, and duplicates and irrelevant articles were removed (Fig. 3). The titles and abstracts of the studies were then screened against the inclusion and exclusion criteria below.

Inclusion criteria

1. Only studies reporting comorbidities and patient’s outcome were included.

2. Only peer-reviewed journals were included.

Exclusion criteria

1. Literature and/or systematic reviews, letters, comments, case reports, and family-based studies were excluded.

2. Articles not reporting SARS-CoV-2 infections were excluded.

3. Studies not reporting the nature of comorbidity, hospital names, or regions from which study participants were drawn and the period for which the study was conducted were excluded.

### Data extraction.

A data extraction form was developed in MS-Excel, and data were extracted independently by T.T. The data extracted was subjected to screening by W.H.N. and unanimity was reached on disputes after discussion. A third author was not involved, as there were no major disputes in study selection. Data extracted in this study, when available, included the title, published year, number of participants, country, patient’s age and gender, identified comorbidities, and the percentage of infected patients, type of intervention, study type, and patient outcome. In cases of studies with overlapping participants, the study with the longest sampling period was selected.

### Statistical analysis.

Meta-analysis was conducted using pooled studies which reported bivariate analysis. The study aims to identify the association of comorbidity and its exacerbation of the outcome in SARS-CoV-2-infected patients resulting in death. Therefore, univariate analysis is not applicable, as it does not deal with the association. The models used for multivariate analysis, on the other hand, vary greatly in terms of the parameters used to justify its inclusion. Statistical analysis was performed using R, version 4.0.2 (R Foundation for Statistical Computing, Vienna, Austria). We performed a random-effects meta-analysis to account for between- and within-study differences across studies in the meta-analysis. For each random-effects meta-analysis, relative weight, based on the within- and between-study variances was applied. Heterogeneity was assessed based on two approaches, a scale with cutoffs at 25%, 50%, and 75% for low, intermediate, and high inconsistency, respectively, among the study findings, and level of statistical significance, where *P* < 0.05 shows that the true effects vary (104). A nonsignificant *P* value for heterogeneity implies weakness in the effect sizes but could also be related to the sample sizes across the studies analyzed. Results from the meta-analysis were summarized into either natural logarithm of odds ratio (log.OR) and 95% confidence intervals (95% CI) or natural logarithm of hazard ratio (log.HR) and 95% CI depending on the outcome considered.
