# Supplementary material for: Comorbidities in SARS-CoV-2 Patients: a Systematic Review and Meta-Analysis
Source: mBio. 2021 Feb 9;12(1):e03647-20. doi: 10.1128/mBio.03647-20 (PMC7885108; doi:10.1128/mBio.03647-20)
Supplement: TABLE S1 [file mBio.03647-20-st001.docx]

**Table S1.** Characteristics of included studies. Data included in this table, when available, included the title, country, age, number of participants, and the type of analysis applied.

| **Reference** | **Title** | **Country** | **Age, years**  **Median (IQR)** | **Sample number** | **Univariate Analysis** | **Bivariate Analysis** | **Multivariate Analysis** |
| --- | --- | --- | --- | --- | --- | --- | --- |
| Bhumbra et al | Clinical Features of Critical Coronavirus Disease 2019 in Children | USA | 5 (0.8–16) | 19 | Disease severity: comorbidities p = 0.999 |  |  |
| Vena et al | Clinical characteristics, management and in-hospital mortality of patients with coronavirus disease 2019 in Genoa, Italy | Italy | 71 (60–82) | 317 | Mortality: **Hypertension** p < 0.001 **Cardiovascular disease** p < 0.001, **Diabetes mellitus** p = 0.01**, Chronic kidney disease** p = 0.01**, Chronic obstructive lung disease** p = 0.03**,** solid cancer p = 0.11**,** hematological malignancy p = 0.19**, Neurological disease** p < 0.001 |  | Mortality: **Cardiovascular disease:** OR 2.58, 95% CI (1.07-6.25), p = 0.03 |
| Chhiba et al | Prevalence and characterization of asthma in hospitalized and nonhospitalized patients with COVID-19 | USA | NR | 1526 |  |  | Asthma was not associated with an increased risk of hospitalization. RR 0.96, 95% CI (0.77-1.19), p = 0.71 after adjusting for age, sex, race/ethnicity and comorbidities |
| Wu et al | Risk Factors Associated With Acute Respiratory Distress Syndrome and Death in Patients With Coronavirus Disease 2019 Pneumonia in Wuhan, China | China | NR | 201 |  | ARDS: **Hypertension** (yes vs no) HR 1.82, 95% CI (1.13-2.95), p = 0.01, **Diabetes** (yes vs no) HR 2.34, 95% CI (1.35-4.05), p = 0.002 Deaths: Hypertension HR 1.70, 95% CI (0.92-3.14), p = 0.09 Diabetes HR 1.58, 95% CI (0.80-3.13), p = 0.19 |  |
| Shabrawishi et al | Clinical, radiological and therapeutic characteristics of patients with COVID-19 in Saudi Arabia | Saudi Arabia | ^a^46.1 ^c^(15.3) | 150 | Severe outcome: Comorbidities p < 0.05 Hypertension p = 0.627  Diabetes mellitus p = 0.005  Coronary artery disease p = 0.094  **Renal disease** p = 0.018  Thyroid gland problem (hypothyroidism) p = 0.123  Asthma p = 0.306  **Cancer** p = 0.007  Cerebrovascular accident p = 0.718  COPD p = 0.086  Chronic liver disease p = 0.086 |  |  |
| Wang et al | Cancer patients in SARS-CoV-2 infection: a single-center experience from Wuhan | China | 55 (42-65) | 716 | ^b^Severe events: **Cancer** **patients**: p = 0.012 |  | Cancer OR 6.51, 95% CI (1.72-24.64), p = 0.006 Comorbidities: OR 3.100, 95% CI (1.673-5.744), p < 0.001 |
| Romagnolo et al | Neurological comorbidity and severity of COVID-19 | Italy | 61.5 ± ^c^17.8 ^d^(15-98) | 344 |  | Severe infection: **Neurological disease** OR 5.855 95% CI (3.387–10.122), p < 0.001  **Arterial hypertension** OR 4.236, 95% CI (2.631–6.821), p < 0 .001  **Neoplastic disease** OR 4.575, 95% CI (2.413–8.672), p < 0 .001  **Diabetes** OR 2.950, 95% CI (1.528–5.696), p = 0.001  **Chronic obstructive pulmonary disease** OR 2.497, 95% CI (1.291–4.830), p = 0.007  **Moderate-to-severe renal failure** OR 5.835, 95% CI (2.231–10.088), p = 0.001 | Severe infection: **neurological diseases** OR 2.305, 95% CI (1.053–5.046), p = 0.012, **neoplastic diseases** (p = 0.039), **arterial hypertension** (p = 0.045) |
| Martos et al | Comorbidity and prognostic factors on admission in a COVID-19 cohort of a general hospital | Spain | NR | 96 | Bivariate analysis; Mortality: **Presence of comorbidities** p = 0.004 |  | Mortality: **Cardiopathy** OR 13.162, 95% CI (2.583- 67.073), p = 0.002 |
| Freites Nunez et al | Risk factors for hospital admissions related to COVID-19 in patients with autoimmune inflammatory rheumatic diseases | Spain | 59.88 ^c^(14.90) | 123 |  | Hospital admission: **Hypertension** OR 3.64, 95% CI (1.65–8.06), p = 0.001  Dyslipidaemia OR 1.82, 95% CI (0.77–4.32), p = 0.17  **Depression** OR 0.14, 95% CI (0.01–1.18), p = 0.07  **Diabetes mellitus** OR 5.15, 95% CI (1.5–16.8), **p = 0.007**  Heart disease OR 2.9, 95% CI (0.93–9.09), p = 0.06  Vascular disease OR 4.18, 95% CI (0.81–21.64), p = 0.09  Liver disease OR 1.76, 95% CI (0.37–8.22), p = 0.47  **Lung disease (ILD/COPD)** OR 3.32, 95% CI (1.17–9.45), p = 0.02  Cancer OR 5.4, 95% CI (0.58–50.1), p = 0.13  Thyroid disease OR 0.48, 95% CI (0.15–1.47), p = 0.2 | Hospital admission: **AIRD** (systemic autoimmune conditions vs chronic inflammatory arthritis) OR 3.55, 95% CI (1.30–9.67), p = 0.01, Comorbidities OR 1.82, 95% CI (0.69–4.80), p = 0.22 |
| Soares et al | Risk Factors for Hospitalization and Mortality due to COVID-19 in Espírito Santo State, Brazil | Brazil | NR | 10713 |  | Hospitalization: **Cardiovascular diseases** OR 3.15, 95% CI (2.77–3.57), p < 0.001 **Diabetes** OR 3.36, 95% CI (2.88–3.91), **p < 0.001**  **Kidney diseases** OR 7.43, 95% CI (5.20–10.56, p < 0.001  **Obesity** OR 2.04, 95% CI (1.64–2.52), p < 0.001**Pulmonary diseases** OR 2.38, 95% CI (1.90–2.95), p < 0.001 **Smoking** OR 5.12, 95% CI (3.82–6.81), p < 0.001    Death: **Cardiovascular diseases** OR 2.02, 95% CI (1.59–2.57), p < 0.001 **Diabetes** OR 1.88, 95% CI (1.43–2.47), p < 0.001 **Kidney diseases** OR 2.51, 95% CI (1.48–4.35), p = 0.001) **Pulmonary diseases** OR 1.77, 95% CI (1.19–2.62), p = 0.004) | Hospitalization: **Cardiovascular diseases** OR 1.30, 95% CI (1.11–1.53), **p = 0.001**  **Diabetes** OR 1.34, 95% CI (1.10–1.61), **p = 0.003**  **Kidney diseases** OR 2.41, 95% CI (1.59–3.66), **p < 0.001**  **Obesity**  OR 1.74, 95% CI (1.35–2.23), p< 0.001  Pulmonary diseases OR 1.46, 95% CI (1.12–1.90), p= 0.005  **Smoking** OR 2.91, (2.04–4.12), p < 0.001    Death: Cardiovascular diseases OR 1.26, 95% CI (0.95–1.67), p = 0.109 **Kidney diseases** OR 1.68, 95% CI (0.94–3.09), p = 0.086 |
| Salacup et al | Characteristics and clinical outcomes of COVID‐19 patients in an underserved‐inner city population: A single tertiary center cohort | USA | 66 (58‐76) | 242 |  |  | Mortality: Death: COPD and asthma OR 0.471, 95% CI (0.156‐1.428), p = 0.184  Diabetes mellitus OR 1.450, 95% CI (0.672‐3.129), p = 0.344  Hypertension OR 0.954, 95% CI (0.375‐2.426), p = 0.922  Heart failure OR 2.125, 95% CI (0.835‐5.410), p = 0.114  Cirrhosis OR 2.605, 95% CI (0.389‐17.428), p = 0.324  Chronic kidney disease OR 0.806, 95% CI (0.317‐2.048), p = 0.651 |
| Derespina et al | Clinical Manifestations and Outcomes of Critically Ill Children and Adolescents with Coronavirus Disease 2019 in New York City | USA | 15.0 (9.0-19.0) | 70 |  |  | Time to PICU discharge: Any comorbidity AHR 1.29, 95% CI (0.68-2.45), p = 0.4377 time to hospital discharge: Any comorbidity AHR 0.98, 95% CI (0.51-1.91), p = 0.9620 |
| Chachkhiani et al | Neurological complications in a predominantly African American sample of COVID-19 predict worse outcomes during hospitalization | USA | 60 ^c^(15) | 250 |  | Length of hospital stay: **Hypertension** HR 1.4, 95% CI (1.0-2.0), p = 0.03  Smoker HR 0.7, 95% CI (0.4- 1.1), p = 0.1  Epilepsy HR 1.8, 95% CI (0.8-4.5), p = 0.2  Diabetes HR 1.1, 95% CI (0.9- 1.5), p = 0.4  BMI HR 1.0, 95% CI (1.0- 1.0), p = 0.6  Migraine HR 1.4, 95% CI (0.4- 5.2), p = 0.6  Asthma HR 1.1, 95% CI (0.7- 1.6), p = 0.8  Prior CVA HR 1.0, 95% CI (0.7- 1.5), p = 1.0    Death: Epilepsy HR 2.6, 95% CI (0.9-7.7), p = 0.1  Hypertension HR 1.7, 95% CI (0.8-3.7), p = 0.2  Smoker HR 0.5, 95% CI (0.3-1.9), p = 0.3  Diabetes HR 1.1, 95% CI (0.6-1.8), p = 0.8  Asthma HR 1.2, 95% CI (0.6-2.4), p = 0.6  Migraine HR 2.0, 95% CI (0.2-20.4), p = 0.6  BMI HR 1.0, 95% CI (1.0- 1.0), p = 0.8  Prior CVA HR 1.0, 95% CI (0.5-2.1), p = 1.0 Intubation: Asthma HR 1.5, 95% CI (0.7-3.0), p = 0.3  Hypertension HR 1.4, 95% CI (0.7- 2.9), p = 0.4  Prior CVA HR 0.7, 95% CI (0.3- 1.6), p = 0.4  Smoker HR 0.6, 95% CI (0.2- 1.9), p = 0.5  Diabetes HR 1.2, 95% CI (0.7- 2.0), p = 0.6  BMI HR 1.0, 95% CI (1.0-1.0), p = 0.8  Epilepsy HR 1.3, 95% CI (0.3- 4.9), p = 0.8  Migraine HR 1.2, 95% CI (0.1- 13.2), p = 0.9 |  |
| Bartoletti et al | Development and validation of a prediction model for severe respiratory failure in hospitalized patients with SARS-CoV-2 infection: a multicentre cohort study (PREDI-CO study) | Italy | 65.7 ^c^(15) | 1113 |  |  | Severe respiratory failure: **Obesity** OR 4.62, 95% CI (2.78-7.70), p < 0.001 |
| Parra-Bracamonte et al | Clinical characteristics and risk factors for mortality of patients with COVID-19 in a large data set from Mexico | Mexico | 44 (33–56) | 331298 |  |  | Mortality: **Hypertension** OR 1.243, 95% CI (1.194–1.294), p < 0.0001  **Obesity** OR 1.223, 95% CI (1.173–1.275), p < 0.0001  **Diabetes** OR 1.288, 95% CI (1.237–1.341), p < 0.0001  Cardiopathy OR 0.976, 95% CI (0.894–1.064), p = 0.9069  **COPD** OR 1.261, 95% CI (1.150–1.383), p < 0.0001  **Asthma** OR 0.949, 95% CI (0.832–1.082), p < 0.0306  **Immunosuppressed**  OR 1.211, 95% CI (1.078–1.359), p < 0.0001  **CKD** OR 1.802, 95% CI (1.657–1.960), p < 0.0001 |
| Yang et al | Effect of hypertension on outcomes of adult inpatients with COVID-19 in Wuhan, China: a propensity score–matching analysis | China | NR | 226 | Univariate; Mortality: **Hypertension** p < 0.001  **Type 2 diabetes mellitus** p = 0.012  Coronary heart disease p = 0.255 |  | Death: **Hypertension** HR 3.317, 95% CI (1.709–6.440), p < 0.001 |
| Pablos et al | Clinical outcomes of hospitalized patients with COVID-19 and chronic inflammatory and autoimmune rheumatic diseases: a multicentric matched cohort study | Spain | NR | 456 |  | Poor Outcome: **Connective tissue disorder** OR 1.64, 95% CI (1.02 -2.66), p = 0.042 **Obesity** OR 1.78, 95% CI (1.13 -2.81), p = 0.013 **Diabetes** OR 1.81, 95% CI (1.11- 2.95), p = 0.018 **Hypertension** OR 2.60, 95% CI (1.72- 3.94), p < 0.001 **Heart failure** OR 3.49, 95% CI (2.21- 5.51), p < 0.001,  **Lung disease** OR 2.15, 95% CI (1.34- 3.45), p = 0.001 | Poor outcome: **Connective tissue disorder** OR 1.82, 95% CI (1.00 -3.30), p = 0.050, Obesity OR 1.47, 95% CI (0.86 -2.51), p = 0.164  Diabetes OR 0.82, 95% CI (0.46 -1.46), p = 0.493  Heart failure OR 1.57, 95% CI (0.93 -- 2.66), p = 0.092 |
| He et al | Clinical Characteristics and Outcomes of Patients with Severe COVID-19 and Chronic Obstructive Pulmonary Disease (COPD) | China | 65 (50–77) | 336 | Mortality: **COPD** p < 0.001  **Essential hypertension** p = 0.018  **Diabetes** p = 0.001  **Cardiovascular disease** p < 0.001  Cerebrovascular disease p = 0.265  Chronic kidney disease p = 0.903  Chronic liver disease p = 0.824  Malignancy p = 0.798  Autoimmune disease p = 0.549 |  | Mortality: **COPD** adjusted for age and sex HR 2.75, 95% CI (1.01- 5.67), p = 0.018), **COPD** adjusted for hypertension and cardio-cerebrovascular disease HR 1.98; 95% CI (0.59-- 3.44), p = 0.037) |
| Dorcherty et al | Features of 20 133 UK patients in hospital with covid-19 using the ISARIC WHO Clinical Characterisation Protocol: prospective observational cohort study | England, Wales, and Scotland | 72.9 (58.0-82.0) | 20 133 |  |  | Death: **Chronic cardiac disease** HR 1.16, 95% CI (1.08-1.24), p < 0.001, **Chronic pulmonary disease** HR 1.17, 95% CI (1.09-1.27), p < 0.001, **Chronic kidney disease** HR 1.28, 95% CI (1.18-1.39), p < 0.001 Diabetes HR 1.06, 95% CI (0.99-1.14), p = 0.087, **Obesity** HR 1.33(1.19-1.49) p < 0.001, **Chronic neurological disorder**  HR 1.17, 95% CI (91.06-1.29), p = 0.001, **Dementia** HR 1.40, 95% CI (1.28-1.52), p < 0.001, **Malignancy** HR 1.13, 95% CI (1.02-1.24), p = 0.017, Moderate/severe **liver disease** HR 1.51, 95% CI (1.21-1.88), p < 0.001 |
| Smith et al | Identifying Patients at Greatest Risk of Mortality due to COVID-19: A New England Perspective | USA | ^a^66.86 | 346 | Bivariate analysis: Mortality: **Hypertension** p = 0.037 Hyperlipidemia p = 0.429  **Diabetes** p = 0.004  **Cardiovascular disease** p = 0.012  Neurological disease p = 0.373  **CKD** p = 0.016  **COPD** p = 0.001  Cancer p = 0.037  Asthma p = 0.246  Hypothyroid p = 0.128 |  | Mortality: **COPD** RR 1.41 95% CI (1.06–1.88), p = 0.019  **Diabetes** RR 1.43, 95% CI (1.07–1.92), p = 0.016  **Hyperlipidemia** RR 0.75, 95% CI (0.57–0.98), p = 0.032 |
| Giorgi Rossi et al | Characteristics and outcomes of a cohort of COVID-19 patients in the Province of Reggio Emilia, Italy | Italy | NR | 2653 |  |  | Hospitalization  **COPD** HR1.9, 95% CI (1.4–2.5)  Coronary heart disease HR 1.3, 95% CI (1.0–1.7)  Dementia HR 1.2, 95% CI (0.9–1.8)  Diabetes HR 1.5, 95% CI (1.3–1.9)  **Chronic kidney disease** HR 1.9, 95% CI (1.3–2.9)  Cancers HR 1.4, 95% CI (1.1–1.7)  Hypertension HR 1.4, 95% CI (1.2–1.6)  Obesity HR 1.4, 95% CI (0.9–2.0)  **Heart failure** HR 1.6, 95% CI (1.2–2.1)  Arrhythmia HR 1.5, 95% CI (1.2–1.9)  Dyslipidaemia HR 1.3, 95% CI (0.99–1.69)  Vascular disease HR 1.2, 95% CI (0.8–1.8)  Death  COPD HR 1.1, 95% CI (0.7–1.7)  **Coronary heart disease** HR 1.7, 95% CI (1.2–2.5)  **Dementia** HR 1.8, 95% CI (1.1–2.8)  **Diabetes** HR 1.6, 95% CI (1.1–2.2)  Chronic kidney disease HR 1.5, 95% CI (0.9–2.6)  Cancers HR 1.4, 95% CI (1.0–2.0)  Hypertension HR 1.6, 95% CI (1.2–2.1)  Obesity HR 1.3, 95% CI (0.6–2.9)  **Heart failure** HR 2.3, 95% CI (1.6–3.2)  **Arrhythmia** HR 1.8, 95% CI (1.3–2.5)  Dyslipidaemia HR 1.4, 95% CI (0.9–2.2) |
| Lee et al | Epidemiological and clinical characteristics of coronavirus disease 2019 in Daegu, South Korea | South Korea | 52.10 ^c^(18.29) | 694 | Severe disease: **Hypertension** p = 0.000  **Diabetes mellitus** p = 0.000  Coronary artery disease p = 0.137  **Stroke** p = 0.012  Asthma p = 0.319  Chronic obstructive pulmonary disease p =0.193  Old tuberculosis p = 1.000  Heart failure p = 0.228  **Chronic kidney disease** p = 0.008  Liver disease p = 0.063  Thyroid disease p = 0.674  **Dementia** p = 0.037  Cancer p = 1.000 |  |  |
| Sousa et al | Mortality and survival of COVID-19 | Brazil | NR | 2070 |  |  | Death: Cardiovascular disease IRR 9.5, 95% CI (5.4-17.0), p < 0.001  Diabetes IRR 1.5, 95% CI (1.0-2.30), p = 0.08  Hematologic disease IRR 0.7, 95% CI (0.2-3.00), p = 0.66  **Neurologic disease** IRR 3.7, 95% CI (1.8-7.9), p < 0.001  Obesity IRR 3.5, (0.7-18) p = 0.13  Pneumopathies IRR 2.0, 95% CI (1.1-3.9), p = 0.04 Renal disease IRR 0.6, (0.3-1.2), p = 0.12 |
| Chaudhry et al | Clinical characteristics and outcomes of COVID‐19 in solid organ transplant recipients: A case‐control study | USA | NR | 135 |  |  | Death: Diabetes OR 1.48, 95% CI (0.55-3.93), p = 0.44, Chronic kidney disease OR 0.84, 95% CI (0.29-2.49), p = 0.76 Composite outcome: **Diabetes** OR 4.07, 95% CI (1.52-10.89), p = 0.005  Chronic kidney disease OR 1.32, 95% CI (0.47-3.65), p = 0.60 |
| van Gerwen et al | Risk factors and outcomes of COVID‐19 in New York City; a retrospective cohort study | USA | NR | 3703 |  |  | Hospitalization: the following were associated with increased risk of hospitalization  **Atrial fibrillation** OR 1.49, 95% CI (1.03‐2.14), **Cerebrovascular accident/transient ischemic attack** OR 2.25,95% CI (1.42‐3.58) **Dementia** OR 3.60,95% CI (2.12‐ 6.09) **Diabetes** OR 1.71, 95% CI (1.40‐2.09) **Chronic kidney disease** OR 1.88,95% CI (1.33‐2.66) and **COPD** OR: 2.27; 95% CI (1.41‐3.65)  Mechanical ventilation in hospitalized patients:  **Diabetes** was associated with an increased risk of mechanical ventilation (OR: 1.35; 95% CI: 1.08‐1.69). Dementia was associated with a decreased risk of mechanical ventilation (OR: 0.52; 95% CI: 0.34‐0.80)  Mortality in hospitalized patients:  **Congestive heart failure** (OR: 1.47; 95% CI: 1.06‐2.02) and **dementia** (OR: 2.03; 95% CI: 1.46‐2.83) were associated with increased odds of death. The **presence of more than two comorbidities** was associated with increased odds of death compared with no comorbidities (OR: 1.90; 95% CI: 1.35‐2.68)  Mortality in hospitalized patients on mechanical ventilation:  **Dementia** was associated with an increased odd of death (OR: 6.36; 95% CI: 1.29‐31.34). **The presence of more than two** comorbidities was associated with increased odds of death in patients on mechanical ventilation compared with no comorbidities (OR: 2.12; 95% CI: 1.15‐4.03). |
| Garrazino et al | Multicentre Italian study of SARS-CoV-2 infection in children and adolescents, preliminary data as at 10 April 2020 | Italy | 5 2.3 (0.3–9.6) | 168 | The hospitalization rate was similar between children with comorbidities and those without (23/33 vs 87/135, respectively; p = 0.68 |  |  |
| Ciceri et al | Early predictors of clinical outcomes of COVID-19 outbreak in Milan, Italy | Italy | 65 (56–75) | 410 |  | Death: **Presence of comorbidity** HR 3.91, 95% CI (2.26–6.84), p < 0.001  **Hypertension** HR 2.60, 95% CI (1.67–4.05), p < 0.001  Diabetes HR 1.51, 95% CI (0.96–2.05), p = 0.06  **Coronary artery disease** HR 3.21, 95% CI (2.02–5.10), p < 0.001  **Chronic kidney failure** HR 2.75, 95% CI (1.47–4.40), p < 0.001  **Cancer** HR 2.77, 95% CI (1.47–5.22), p = 0.002 | Death: **Coronary artery disease** HR 2.93, 95% CI (1.77–4.86), p < 0.001  **Cancer** HR 2.32, 95% CI (1.15–4.67), p = 0.01 |
| Wang et al | Low high-density lipoprotein level is correlated with the severity of COVID-19 patients: an observational study | China | 45.5 (36.0–60.8) | 228 |  |  | Severe disease: Hypertension HR 1.576, 95% CI (0.593–4.188), p = 0.362  Cardiovascular disease HR 0.888, 95% CI (0.107–7.364), p = 0.912 |
| Graham et al | SARS-CoV-2 infection, clinical features and outcome of COVID-19 in United Kingdom nursing homes | UK | NR | 394 | Mortality: Diabetes p = 0.48  Cardiovascular disease p = 0.0010  Chronic kidney disease p = 0.77  Stroke p = 0.55  Dementia p = 0.35  Lung disease p = 0.51 |  |  |
| Ciardullo et al | Impact of diabetes on COVID-19-related in-hospital mortality: a retrospective study from Northern Italy | Italy | 72 ^c^(14) | 373 | Mortality: Diabetes p = 0.064  Hypertension  p < 0.001  Chronic kidney disease p = 0.014  Tumors p = 0.320  Cardiovascular diseases p = 0.007  COPD p = 0.001 |  | Mortality: **Diabetes** RR 1.559, 95% CI (1.051–2.028), p = 0.030  Hypertension RR 0.977, 95% CI (0.419–1.223), p = 0.927  Chronic kidney disease RR 0.891, 95% CI (0.286–1.311), p = 0.781  Cardiovascular diseases RR 0.916, 95% CI (0.546–1.396), p = 0.712  **COPD** RR 1.821, 95% CI (1.133–2.349), p = 0.019 |
| Garcia-Azorin et al | Neurological Comorbidity Is a Predictor of Death in Covid-19 Disease: A Cohort Study on 576 Patients | Spain | 67.18 ^c^(14.75) | 576 |  | : Mortality: **Hypertension** OR 3.534, 95% CI (2.272–5.495), p < 0.001  **Diabetes** OR 2.129, 95% CI (1.353–3.351), p = 0.001  **Smoking** OR 1.589, 95% CI (1.004–2.514), p = 0.048  **Cardiological disorders** OR 2.955, 95% CI (1.950–4.478), p < 0.001  Pulmonary disorders OR 1.434, 95% CI (0.928–2.217), p = 0.105  **Cancer** OR 1.641, 95% CI (1.001–2.690), p = 0.049  **Chronic neurological disorders** OR 3.961, 95% CI (2.516–6.234), p < 0.001  Immunosuppression OR 1.295, 95% CI (0.405–4.138), p = 0.663 | Mortality: Hypertension OR 1.369, 95% CI (0.806–2.325), p = 0.246  Diabetes OR 1.221, 95% CI (0.710–2.098), p = 0.471  Smoking OR 1.720, 95% CI (0.955–3.096), p = 0.701  Cardiological disorders OR 1.208, 95% CI (0.730-1.999), p = 0.462  Pulmonary disorders OR 0.931, 95% CI (0.543–1.596), p = 0.794  Cancer OR 1.209, 95% CI (0.676–2.162), p = 0.523  **Chronic neurological disorders** OR 1.763, 95% CI (1.014–3.064), p = 0.044 |
| Giacomelli et al | 30-day mortality in patients hospitalized with COVID-19 during the first wave of the Italian epidemic: A prospective cohort study | Italy | NR | 233 |  | Mortality: **Obesity** HR 2.01, 95% CI (1.07−3.81), p = 0.031 | Mortality: **Obesity** HR 3.04, 95% CI (1.42−6.49), p = 0.004 |
| Gutierrez Rodriguez et al | Variables associated with mortality in a selected sample of patients older than 80 years and with some degree of functional dependence hospitalized for COVID-19 in a Geriatrics Service | Spain | 88.3^c^(5.4) | 58 |  | Mortality: Hypertension OR 2.4, 95% CI (0.7- 8.7), p = 0.179  Diabetes OR 0.7, 95% CI (0.2- 2, 2), p = 0.545  Dyslipidemia OR 1.3, 95% CI (0.4- 3.6), p = 0.658  Cancer OR 1.5, 95% CI (0.2- 11.1), p = 0.717  COPD / asthma OR 1.1, 95% CI (0.2- 5.3) p = 0.933  **Chronic kidney disease** OR 3.2, 95% CI (1.1- 9.7), p = 0.033 |  |
| Ebinger et al | Pre-existing traits associated with Covid-19 illness severity | USA | 52.72 ^c^(19.65) | 442 | Illness severity: Obesity p = 0.059  Hypertension p < 0.001  Diabetes mellitus p < 0.001 |  | Illness severity (hospitalization, intensive care, intubation): Obesity OR 1.95, 95% CI (1.11-3.42), p = 0.021  Hypertension OR 1.19, 95% CI (0.71-1.99), p = 0.52  **Diabetes mellitus** OR 1.77, 95% CI (1.03-3.03), p = 0.037 |
| Twigg et al | Mortality Rates in a Diverse Cohort of Mechanically Ventilated Patients With Novel Coronavirus in the Urban Midwest | USA | 59.6 ^c^(15.5) | 242 |  |  | Mortality: Hypertension HR 1.9, 95% CI (0.7–5.2), p = 0.189  Chronic obstructive pulmonary disease HR 1.5, 95% CI (0.6–3.7), p = 0.391  Chronic kidney disease HR 1.0, 95% CI (0.5–2.3), p = 0.942  **End-stage renal disease** HR 5.9, 95% CI (1.3–26.9), p = 0.021 |
| Wu et al | Risk Factors Associated With Long-Term Hospitalization in Patients With COVID-19: A Single-Centered, Retrospective Study | China | 55.0 (40.0–68.5) | 125 | Hospitalization: Hypertension p = 0.160  Type 2 diabetes p = 0.929  Coronary heart disease p = 0.253 |  |  |
| Klang et al | Severe Obesity as an Independent Risk Factor for COVID-19 Mortality in Hospitalized Patients Younger than 50 | USA | NR | 3406 | Mortality: Univariate analysis: Age ≤ 50  Coronary artery disease p = 0.132  Congestive heart failure p = 0.002  Hypertension p = 0.104  **Diabetes mellitus** p = 0.020  Hyperlipidemia p = 0.220  **Chronic kidney disease** p < 0.001  **Cancer** p= 0.014  Smoking p= 0.067 BMI  < 30 kg/m^2^  p = 0.102  30 - 40 kg/m^2^  p = 0.313  **≥ 40 kg/m^2^** p < 0.001  Age > 50  **Coronary artery disease** p < 0.001  **Congestive heart failure** p < 0.001  **Hypertension** p < 0.001  **Diabetes mellitus** p < 0.001  **Hyperlipidemia** p < 0.001  **Chronic kidney disease** p < 0.001  Cancer p = 0.138  Smoking p = 0.168 BMI  < 30 kg/m2 p = 0.269  30 - 40 kg/m2 p = 0.117  ≥ 40 kg/m2 p = 0.532 |  | Mortality: Age ≤ 50  Chronic artery disease OR 0.6, 95% CI (0.2 – 2.1), p = 0.418  **Congestive heart failure** OR 4.0, 95% CI (1.6 – 10.4), p = 0.004  Hypertension OR 0.5, 95% CI (0.2 – 1.1), p = 0.088  Diabetes mellitus OR 1.3, 95% CI (CI 0.7 – 2.6) p = 0.442  Hyperlipidemia OR 0.8, 95% CI (0.3 – 2.10), p = 0.710  **Chronic kidney disease** OR 3.3, 95% CI (1.4 – 7.7), p = 0.006  Cancer OR 2.5, 95% CI (1.0 – 6.5), p = 0.052  Smoking OR 1.7, 95% CI (0.8 – 3.8), p = 0.162  Age > 50  **Chronic artery disease** OR 1.3, 95% CI (1.1 – 1.6), p = 0.006  Congestive heart failure OR 1.0, 95% CI (0.8 – 1.3), p = 0.954  Hypertension OR 1.1, 95% CI (0.9 – 1.3), p = 0.571  **Diabetes mellitus** OR 1.4, 95% CI (1.2 – 1.7), p < 0.001  Hyperlipidemia OR 1.0, 95% CI (0.8 – 1.2) p = 0.898  **Chronic kidney disease** OR 1.7, 95% CI (1.4 – 2.1), p < 0.001  Cancer OR 1.0, 95% CI (0.8 – 1.2), p = 0.936  Smoking OR 1.0, 95% CI (0.8 – 1.2), p = 0.950 |
| Nikpouraghdam et al | Epidemiological characteristics of coronavirus disease 2019 (COVID-19) patients in IRAN: A single center study | Iran | NR | 2964 |  |  | Mortality**: Comorbidity** OR 1.53, Standard error 0.19 (1.04 - 2.24), p = 0.03 |
| Portoles et al | Chronic kidney disease and acute kidney injury in the COVID-19 Spanish outbreak | Spain | 64.2 ^c^(15.6) | 1603 |  | Mortality: Any comorbidity HR 1.20, 95% CI (0.90–1.60) Diabetes HR 1.83, 95% CI (1.34–2.50)  Hypertension/CV HR 1.19, 95% CI (0.89–1.58)  Obesity HR 1.22, 95% CI (0.63–2.33)  Immunosuppression HR 1.48, 95% CI (0.78–2.80)  Chronic kidney disease HR 4.17, 95% CI (3.08–5.66) |  |
| Chen et al | Disease progression patterns and risk factors associated with mortality in deceased patients with COVID-19 in Hubei Province, China | China | 55.0 (36.0‐68.0) | 792 | Mortality: Hypertension p < 0.001  Diabetes p <0.001  COPD p = 0.005  Chronic kidney disease p = 0.049 |  | Mortality: **Number of preexisting comorbidities** ≥2 OR 6.68, 95% CI (2.14‐29.44), p = 0.002 |
| Senkal et al | Association between chronic ACE inhibitor exposure and decreased odds of severe disease in patients with COVID-19 | Turkey | NR | 611 | Severe outcome: Hypertension p < 0.001  Diabetes mellitus p = 0.12  COPD/Asthma p = 0.087  Coronary artery disease p = 0.004  Congestive heart failure p = 0.029  Chronic kidney disease p = 0.009  Solid malignancy p = 0.37  Hematologic malignancy p = 0.59  Smoking p = 0.56 | Severe disease: history of hypertension, CAD, CHF, and CKD were associated with increased odds of severe disease |  |
| Raad et al | Cardiac Injury Patterns and Inpatient Outcomes Among Patients Admitted With COVID-19. | USA | 63 (52–73) | 1020 |  |  | Death: **Cerebrovascular disease** OR 2.5, 95% CI (1.3–5.0), p = 0.008 |
| Kirenga et al | Characteristics and outcomes of admitted patients infected with SARS-CoV-2 in Uganda. | Uganda | 33 (25–43) | 56 | The primary outcomes (admission to ICU, mechanical ventilation or death) did not occur in any patient. |  |  |
| Killerby et al | Characteristics Associated with Hospitalization Among Patients with COVID-19 — Metropolitan Atlanta, Georgia, March–April 2020 | USA | NR | 531 |  |  | Factors predicting Hospitalization: diabetes mellitus OR 3.1, 95% CI (1.7–5.9) smoking OR 2.395% CI (1.2–4.5) obesity OR 1.9 95% CI (1.1–3.3) |
| Lai et al | Characteristics Associated With Out-of-Hospital Cardiac Arrests and Resuscitations During the Novel Coronavirus Disease 2019 Pandemic in New York City | USA | NR | 5325 |  |  | out-of-hospital cardiac arrest resuscitations: **Cardiac disease** OR 0.72, 95% CI (0.61-0.86), p < 0.001  **Hypertension** 0R 1.28, 95% CI (1.09-1.50), p = 0.002  **Diabetes** OR 1.45, 95% CI (1.23-1.71), p < 0.001  Renal disease OR 0.79, 95% CI (0.60-1.03), p = 0.08  **Asthma/COPD** OR 0.78, 95% CI (0.64-0.95), p = 0.02  **Cancer** OR 0.72, 95% CI (0.56-0.92), p = 0.009  **CVA** OR 0.70, 95% CI (0.52-0.94), p = 0.02 |
| Abrishami et al | Clinical and Radiologic Characteristics of COVID-19 in Patients With CKD | Iran | 60.65 ^c^(14.36) | 43 | Mortality: Ischemic heart disease p > 0.05 hypertension p > 0.05, Diabetes mellitus p > 0.05, Asthma p > 0.05, Chronic kidney disease p=>0.05 |  |  |
| Choi et | Clinical characteristics and disease progression in early-stage covid-19 patients in South Korea | South Korea | 29 (24–47) | 293 |  | Disease progression: **Hypertension** OR 3.95, 95% CI (1.64–9.54), p = 0.002  **Diabetes** mellitus OR 8.60, 95% CI (3.34–22.17), p < 0.001  Allergic disease OR 0.17, 95% CI (0.02–1.28), p = 0.085  Chronic lung disease OR 1.58, 95% CI (0.43–5.78), p = 0.491  Peripheral vascular disease OR 0.58, 95% CI (0.07–4.63), p = 0.610    Liver disease OR 1.81, 95% CI (0.20–16.63), p = 0.601  Congestive heart failure OR 3.72, 95% CI (0.66–21.09), p = 0.138  Cerebrovascular disease OR 4.98, 95% CI (0.80–30.88), p = 0.085  Rheumatic disease OR 7.31, 95% CI (0.45–119.59), p = 0.163,    Progression-free survival of COVID-19 patients:  **Hypertension** OR 3.56, 95% CI (1.67–7.58), p = 0.001  **Diabetes mellitus** OR 6.59, 95% CI (3.17–13.69), p < 0.001  Allergic disease OR 0.1782, 95% CI (0.02–1.30), p = 0.089  Chronic lung disease OR 1.473, 95% CI (0.45–4.80), p = 0.521  Peripheral vascular disease OR 0.6158, 95% CI (0.084–4.50), p = 0.633  **Cerebrovascular disease** OR 4.71, 95% CI (1.13–19.62), p = 0.033  Rheumatic disease OR 4.95, 95% CI (0.68–36.20), p = 0.115  Congestive heart failure OR 3.239, 95% CI (0.78–13.49), p = 0.107  Progression free survival: Hypertension HR 3.56, 95% CI (1.67–7.58), p = 0.001  Diabetes mellitus HR 6.59, 95% CI (3.17–13.69), p < 0.001  Allergic disease HR 0.1782, 95% CI (0.02–1.30), p = 0.089  Chronic lung disease HR 1.473, 95% CI (0.45–4.80), p = 0.521  Peripheral vascular disease HR 0.6158, 95% CI (0.084–4.50), p = 0.633  Cerebrovascular disease HR 4.71, 95% CI (1.13–19.62), p = 0.033  Rheumatic disease HR 4.95, 95% CI (0.68–36.20), p = 0.115  Congestive heart failure HR 3.239, 95% CI (0.78–13.49), p = 0.107 |  |
| Suleyman et al | Clinical Characteristics and Morbidity Associated With Coronavirus Disease 2019 in a Series of Patients in Metropolitan Detroit. | USA | 57.5 ^c^(16.8) | 463 | ICU admission: Asthma p = 0.53  Chronic obstructive pulmonary disease p = 0.56  Obstructive sleep apnea p = 0.43  Any obesity p = 0.43  Severe obesity p = 0.06  **Diabetes** p = 0.02  **Hypertension** p = 0.04  **Chronic kidney disease** p < 0.001  End-stage renal disease p = 0.99  Solid organ transplant p = 0.99  Coronary artery disease p = 0.26  Congestive heart failure p = 0.11  Cancer p = 0.04  Tobacco use p = 0.42 |  | ICU admission: **Severe obesity** OR 2.0, 95% CI (1.4-3.6), p = 0.02  **Chronic kidney disease** OR 2.0, 95% CI (1.3-3.3), p = 0.006  Cancer OR 1.9, 95% CI (1.0-3.9,) p = 0.06  Diabetes OR 1.3, 95% CI (0.8-2.2), p = 0.25  Hypertension OR 1.0, 95% CI (0.5-1.8), p = 0.92  Coronary artery disease OR 1.1, 95% CI (0.6-2.0), p = 0.88, Need for mechanical ventilation: **Severe obesity** OR 3.2, 95% CI (1.7-6.0), p < 0.001  **Chronic kidney disease** OR 2.4, 95% CI (1.4-4.2) p = 0.001  **Cancer** OR 2.5, 95% CI (1.2-5.0), p = 0.01  Diabetes OR 1.2, 95% CI (0.7-2.0), p = 0.58  Hypertension OR 0.9, 95% CI (0.5-1.8), p = 0.81  Coronary artery disease OR 1.3, 95% CI (0.7-2.6), p = 0.45  Congestive heart failure OR 0.7, 95% CI (0.3-1.5), p = 0.38  Tobacco use OR 1.1, 95% CI (0.7-1.9), p = 0.66 |
| Li et al | Clinical Characteristics and Outcomes of 74 Patients With Severe or Critical COVID-19 | China | 66 (55-72) | 74 | Mortality: **Hypertension** p = 0.045  Diabetes p = 0.790  **Coronary disease** p = 0.002  Tuberculosis p = 0.883  Chronic liver disease p = 0.489  Malignancy p = 0.489 |  |  |
| Khamis et al | Clinical characteristics and outcomes of the first 63 adult patients hospitalized with COVID-19: An experience from Oman. | Oman | 48 ^c^(16) | 63 | Mortality was associated with those that had **Diabetes mellitus** (20% vs 2.3%; p = 0.032) |  |  |
| Zou et al | Clinical Characteristics and Risk Factors for Disease Severity and Death in Patients With Coronavirus Disease 2019 in Wuhan, China. | China | 65(55.0–71.5) | 121 |  | Severe disease  **Cardiovascular and cerebrovascular diseases** HR 2.54, 95% CI (1.41–4.58), p = 0.002  **Any comorbidity** HR 4.53, 95% CI (1.78–11.55), p = 0.002  Death:  Cardiovascular and cerebrovascular diseases HR 2.79, 95% CI (0.80–9.71), p = 0.106  **Any comorbidity** HR 7.81, 95% CI (1.02–59.86), p = 0.048 |  |
| Sun et al | Clinical features of patients with coronavirus disease 2019 from a designated hospital in Beijing, China | China | 44.0 (34.0 - 56.0) | 55 | Hospitalized: **Any comorbidity** p = 0.044  **Hypertensio**n p = 0.014  Diabetes p = 0.550  Respiratory diseases p = 0.354  Thyroid disease p = 0.489  Chronic liver disease p = 0.489  Chronic kidney disease p = 0.339  Cardiovascular diseases p = 0.339 |  |  |
| Aggarwal et al | Clinical and Epidemiological Features of SARS-CoV-2 Patients in  SARI Ward of a Tertiary Care Centre in New Delhi | India | 54.5 (46.25 - 60) | 32 | ^e^Primary composite endpoint: **Co-morbidities (at least 1)** p= 0.03  **Co-morbidities (at least 2)** p = 0.02  Hypertension p = 0.03  **Diabetes mellitus** p = 0.003  Heart disease p = 0.58  CVA p = 0.19  Chronic chest condition p = 0.61  H/O PTB p = 0.26  COPD p = 0.26  Asthma p = 0.71  ILD p = 0.19  Hypothyroidism p = 0.71 |  |  |

Progression-free survival (PFS) was defined as the duration of time over which patients with COVID-19 remained stable during their hospitalization. IQR = interquartile range, NR = data not reported

*The primary outcome was a composite outcome, ‘severe COVID-19’, including death, intensive care unit admission, intratracheal intubation or serious COVID-19 complications

^a^Mean age

^b^Severe events were defined as admission to ICU, the use of mechanical ventilation, or death

^c^Standard deviation

^d^Range

^e^Primary composite endpoint was admission to an intensive care unit (ICU), the use of mechanical ventilation or death

AIRD, Autoimmune rheumatic disease; ARDS, Acute Respiratory Distress Syndrome; CAD, Coronary Artery Disease; CHF, Congestive Heart Failure; CKD, Chronic Kidney Disease; COPD, Chronic Pulmonary Disease; CVA, Cerebrovascular Accident; ICU, Intensive Care Unit; ILD, Interstitial lung disease; BMI, Body Mass Index; CI, Confidence Interval; HR, Hazard Ratio; OR, Odds Ratio; RR, Relative Risk.
